# Interactions of gender inequality and parental discipline predicting child aggression in low‐ and middle‐income countries

**DOI:** 10.1111/cdev.14152

**Published:** 2024-08-12

**Authors:** Kaitlin P. Ward, Andrew C. Grogan‐Kaylor, Julie Ma, Garrett T. Pace, Shawna J. Lee, Pamela E. Davis‐Kean

**Affiliations:** ^1^ School of Social Work, Department of Psychology University of Michigan Ann Arbor USA; ^2^ School of Social Work University of Michigan Ann Arbor Michigan USA; ^3^ School of Social Work University of Michigan‐Flint Flint Michigan USA; ^4^ School of Social Work University of Nevada, Las Vegas Las Vegas Nevada USA; ^5^ Department of Psychology University of Michigan Ann Arbor Michigan USA

## Abstract

Children in low‐ and middle‐income countries (LMICs) are disproportionately at risk of not meeting their developmental potential. Parental discipline can promote and hinder child outcomes; however, little research examines how discipline interacts with contextual factors to predict child outcomes in LMICs. Using data from 208,156 households with children between 36 and 59 months (50.5% male) across 63 countries, this study examined whether interactions between gender inequality and discipline (shouting, spanking, beating, and verbal reasoning) predicted child aggression. Results showed aggression was higher in countries with high gender inequality, and associations between discipline and child aggression were *weaker* in countries where gender inequality was *higher*. Improvements in country‐level gender parity, in addition to parenting, will be necessary to promote positive child outcomes in LMICs.

**Abbreviations**
GDP: Gross Domestic Product
GII: Gender Inequality Index
LMICs: low‐ and middle‐income countries
MICS: Multiple Indicator Cluster Surveys
OECD: Organisation for Economic Co‐operation and Development
SDG: Sustainable Development Goal
SSA: sub‐Saharan African
UNDP: United Nations Development Programme
UNICEF: United Nations Children's Fund
WHO: World Health Organization

Children learn the foundations of how to function in society by learning how to regulate and control their behaviors (Moffitt et al., 2011). Parental discipline, which is defined as the verbal or behavioral means by which caregivers control or correct undesired child behavior, is one of the primary ways that children learn the rules and values of their culture and larger society (Lansford, 2019). For example, parental modeling, promotion of correct behavior, and explaining why misbehaviors were wrong (i.e., verbal reasoning), promote children's moral development and can prevent child misbehavior from occurring (Lansford, 2019; Pinquart & Fischer, 2021). Further, helping children understand desirable behavior promotes empathy and positive peer relations (Krevans & Gibbs, 1996). On the other hand, correcting a child's behavior using parental physical aggression, such as slapping, hitting, and yelling, has been shown to harm children's cognitive development and increase child aggressive and antisocial behaviors (Gershoff & Grogan‐Kaylor, 2016; MacKenzie et al., 2013; Straus et al., 1998; Ward et al., 2021). Lack of self‐control as exhibited by childhood behavior problems can then relate to poor adult outcomes including spending more time in the penal system (Koepp et al., 2023). Thus, it is important to understand how lack of self‐control may develop not only by examining the contexts in which children develop in both at the proximal parenting level but also to the broader contexts of culture and values that the parents are responding to when either choosing to model positive (e.g., empathy) or negative (e.g., harsh punishment) behavior. The relationship between parenting and child outcomes is especially important to examine within a child's formative years, which researchers and several international organizations define as the first 5 years of life (Thompson, 2001). This age range is considered critical for brain development and the formation of neural connections (Cao et al., 2017), self‐regulation (Montroy et al., 2016), and behavioral and social development (Gardner & Shaw, 2008). Reflecting the salience of this developmental stage, the United Nations Sustainable Development Goals (SDG) include a dedicated indicator (SDG 4.2.1) that measures whether children under 5 years are developmentally on track in learning, health, and psychosocial well‐being (United Nations, 2024), which also encompasses an assessment of aggressive behavior. Aggressive behavior during early childhood can have a substantial negative impact on children's developmental trajectories. Longitudinal studies have shown that aggression exhibited during children's formative years is associated with higher risk of chronic and serious violence in adolescence and adulthood (Broidy et al., 2003; Tremblay et al., 2004). Understanding the factors associated with child aggression at both the micro and macro contexts can guide the development of preventive interventions during early childhood.

Most psychological research and parenting research comes from samples based in industrialized countries, especially the United States (Henrich et al., 2010). Although parenting in low‐ and middle‐income countries (LMICs) is much less commonly studied (Draper et al., 2022), research suggests that the mechanisms linking parenting practices to child well‐being are similar in LMIC contexts. For example, in one study that used MICS data from 39 LMICs to examine the influence of proximal and distal microsystems to child development of over 100,000 children aged 3–5 years, negative parenting, including physically aggressive parenting strategies, ranked high among a list of 18 predictors in predicting less optimal early childhood outcomes (Bizzego et al., 2022). Indeed, cognitive caregiving and physically violent discipline were the practices that exerted the most influence on on‐track and off‐track child development in five early childhood development index domains. Another study of the MICS data, using data from over 150,000 children and focusing on cognitive and socio‐emotional caregiving practices, found that these caregiving practices were consistently associated with overall better outcomes for early childhood development (Bornstein et al., 2022). Both these studies, and indeed numerous other studies using MICS data, validate developmental theories attesting to the importance of caregiving practices and behaviors as they relate to socio‐emotional and cognitive child development outcomes, even in LMIC contexts with high levels of socioeconomic disadvantage.

## Gender inequality in LMICs

LMICs are rich in cultural, population, and resource diversity, and also exhibit some common challenges. Gender inequality is defined as the lack of equal rights, resources, opportunities, or protection between men and women, or between boys and girls (UNICEF, 2019). Gender inequality gaps favoring men are systematically larger in LMICs (Klasen, 2019). Prior studies involving economically advanced countries and LMICs demonstrated the associations between gender inequality and negative child outcomes including child homicide rates and maltreatment (Butchart & Engström, 2002; Fiala & LaFree, 1988; Klevens & Ports, 2017). While gender inequality tends to be more pronounced in LMICs on average, there are also many LMICs that are making impressive advances toward gender equality and female empowerment. For example, Rwanda ranks number six in overall gender parity according to the World Economic Forum (2015), and Burundi, Mozambique, and Tanzania rank in the top 50. In these countries, there are many female members of parliament, progressive policies that increase wage equality, and high female labor force participation (World Economic Forum, 2015). It is possible that such gender parity advancements in LMICs significantly improve both parenting and child development outcomes.

The World Health Organization (WHO) has warned that gender inequality is a salient risk factor for child maltreatment (Runyan et al., 2002). Indeed, a recent study examining over 50 LMICs found that gender inequality was associated with greater odds of child physical abuse and neglect (Klevens & Ports, 2017). In settings with high gender inequality, women may experience more stress due to inequitable treatment and lack of opportunities, and may be more likely to resort to aggressive parenting and harsher forms of punishment (Fiala & LaFree, 1988; Klevens & Ports, 2017). In addition, disempowered individuals, particularly women, are exposed to higher levels of interpersonal violence (Yodanis, 2004), which may hinder their ability to protect children from aggressive forms of discipline or abuse (Gartner, 1990). To date, the joint effects of gender inequality (or gender parity) *and* parenting on child outcomes have not been examined in LMICs.

Prior research has emphasized that gender inequality is closely linked with economic opportunity, and that increasing gender parity is an essential component of durable economic growth (Dollar & Gatti, 1999; Klasen, 2002). The Organisation for Economic Co‐operation and Development (OECD) estimates that a 50% increase in gender parity would be associated with a 6% increase in Gross Domestic Product (GDP) by 2030 (OECD, 2023). Further, a recent study that included a sample of 105 developing countries found that gender equality in education was particularly important to economic growth among sub‐Saharan African (SSA) countries, and in non‐SSA developing countries, a greater percentage of women in parliament was highly associated with economic growth (as measured by the GDP) (Lin & Dong et al., 2021). Therefore, in attempting to examine the effects of gender inequality on child outcomes, GDP is an important factor to include in quantitative analyses.

## Theoretical frameworks

An ecological perspective (Bronfenbrenner, 2004) often conceptualizes the environment of a family or of a child as a series of nested circles. The inner circle, or microsystem, consists primarily of the family, the mesosystem consists of larger environments such as the neighborhood or community, while the macrosystem refers to larger social systems such as the social structure with its unequal distribution of privileges and oppressions. Typically, for considerations of child development, the microsystem is considered to be a more proximal influence while the larger systems such as the macrosystem are considered to be more distal (Woolley & Grogan‐Kaylor, 2006). The integrative model (García Coll et al., 1996) reconceptualizes the ecological model. One aspect of the integrative model suggests that disadvantages stemming from the macrosystem may be particularly salient—that is, less distal and more proximal—for families that are marginalized by the larger social system, such as families of color or families with low income. Our argument is that, across LMICs, gender discrimination represents a kind of disadvantage that affects the amount of influence parents and caregivers can have on child socio‐emotional development.

A second tradition of theorizing has attempted to discern common elements in attitudes and values across cultures. Particularly notable in this regard has been the work of Inglehart and colleagues (Inglehart & Welzel, 2005) based on multi‐country research from the World Values Survey (Inglehart et al., 2022). Based upon factor analytic work, Inglehart and colleagues hypothesize two broad underlying dimensions of cultural *attitudes* and *values* (Inglehart & Welzel, 2005). Conceptually and statistically, these two dimensions are correlated; yet at the same time, distinct. The two identified dimensions are “traditional values versus secular‐rational values” and “survival values versus self‐expression values” (Inglehart & Welzel, 2005; Kaasa, 2021). Traditional values emphasize the importance of religion, family, authority, and nationalism, while secular‐rational values center on the opposite (Inglehart & Welzel, 2005). Survival values center on physical and economic security, while self‐expression values emphasize high individual autonomy, prioritize tolerance of human diversity, and support gender equality (World Values Survey, 2023).

While the two dimensions encompass broad clusters of traits, Inglehart and Welzel (2005) suggest that the continuum of “survival vs. self‐expression” is most relevant to questions of gender equality. Inglehart and others posit that as countries move further along this continuum toward self‐expression, support for gender equality increases, and people are less constrained by gender roles and sexual norms (Inglehart & Oyserman, 2004; Inglehart & Welzel, 2005). We build upon and extend this thinking by suggesting that, in contexts where gender inequality is low, parenting might have more salience, and more effect on child outcomes; however, in contexts where gender inequality is high, individual parenting decisions might matter less for children's outcomes.

When a child experiences aggressive discipline in the home, and enters an environment that promotes strict adherence to inequitable gender norms, children may exhibit even more aggressive behavior than children living in more equitable societies. For example, when children are exposed to male‐to‐female violence in the home through domestic violence toward their female caregiver, or exposed to harsh punishment from their male caregiver, this role models both gender inequality and a power imbalance in the parent–child relationship (Bandura, 1989). Such role modeling of using violence to reinforce gender inequality and parental power may contribute to children's aggressive behavior (Bandura, 1977; Ma et al., 2022). On the other hand, when parents model nonviolent approaches to misbehavior in the home in an environment where gender parity is reinforced, there may be benefits of such micro–macro interactions, with nonaggressive discipline in the home and more equitable opportunities for men and women in the environment being beneficial to child cognitive and social outcomes (Efevbera et al., 2017; Ward et al., 2022).

According to the bioecological model (Bronfenbrenner, 1999), the power of the microsystem (i.e., parenting) to actualize children's potentials should be greater when inequality is *higher* (see Proposition II in Bronfenbrenner & Morris, 2006). However, when considering theories that emphasize the salience of distal and environmental factors on the health of minority and oppressed populations (see García Coll et al., 1996; Inglehart & Oyserman, 2004), we argue that, when gender inequality is high in a particularly disadvantaged context, the microsystem may provide less of an opportunity to buffer children from deleterious socio‐emotional outcomes, even after controlling for relevant child and household characteristics.

Extant studies have demonstrated positive associations between physically aggressive parenting and child aggression (Grogan‐Kaylor et al., 2021; Ma et al., 2022), negative associations between nonaggressive discipline and child aggression (Ward et al., 2023), and positive associations between gender inequality and aggressive parenting (Klevens & Ports, 2017; Ma et al., 2022). However, the concurrent, joint effects of parenting behaviors and gender inequality on child aggression remain unknown. Therefore, this study examines whether the relationships between physically aggressive discipline behaviors, psychologically aggressive discipline, and nonaggressive discipline and child aggression in the household vary by country‐level gender inequality. Using a confirmatory approach, we hypothesized that the relationship between discipline and child aggression (positive relations for aggressive discipline, and negative relations for nonaggressive discipline) would be weaker in contexts where gender inequality was higher.

## METHOD

### Study design and procedures

This study used publicly available data from the United Nations Children's Fund (UNICEF) Multiple Indicator Cluster Surveys (MICS). For almost 30 years, MICS have collected data from over 100 LMICs to assess the well‐being of women and children. MICS data were collected using multistage cluster sampling, where households were randomly selected for participation within clusters. The UNICEF MICS researchers used representative sampling procedures within each cluster, making survey results comparable across countries. We used MICS rounds 4 (MICS4) and 5 (MICS5), which occurred between 2009–2013 and 2012–2017, respectively. The dataset included publicly released survey responses as of July 2020, which included 858,398 children reported by respondents (MICS4: 364,941; MICS5: 493,457) across 642,541 households in 66 countries.

Within each sampling area, fieldwork teams conducted in‐person interviews with the head of the household. If the head of the household was unavailable at the time of the interview, a spouse of the head‐of‐household or the child's caregiver was interviewed. A “reference” child—between the ages of 2 and 17 years for MICS4, and 1–17 years for MICS5—within the household was chosen via a random number table for the discipline items. After the household survey was completed, the mother or primary caregiver of each child in the household between 36 and 59 months (i.e., a “focal” child) completed a survey with questions on socio‐emotional development. The analytic sample was restricted to households with focal children between 36 and 59 months (i.e., approximately 27.4% of the original sample), as child aggression was only measured for children between 36 and 59 months. Further, participants who skipped all discipline questions were removed from the sample (dropping 2.74% of the sample), and duplicate households were also removed. The final sample size consisted of 208,156 responses across 63 countries. The University of Michigan Institutional Review Board deemed these analyses of de‐identified data exempt from oversight.

### Measures

#### Discipline behaviors

Discipline behaviors toward the reference child were measured using the UNICEF‐modified version of the Parent–Child Conflict Tactics Scales (Straus et al., 1998). All discipline indicators were dichotomous (0 = *no*, 1 = *yes*). Respondents were given the prompt: “Adults use certain ways to teach children the right behavior or to address a behavior problem. I will read various methods that are used. Please tell me if you, or any other adult in your household, has used this method with [child] in the past month.” Psychological aggression was measured with the item: “Shouted, yelled at or screamed at [child].” Physical aggression was measured with two separate items: “Spanked, hit or slapped [child] on the bottom with a bare hand,” and “Beat [child] up, that is, hit [child] over and over as hard as one could.” Verbal reasoning was measured with the item: “Explained why [child]'s behavior was wrong.”

#### Child aggression

To measure child aggression, respondents were asked, “Does [child] kick, bite, or hit other children or adults?” (0 = *no*, 1 = *yes*).

#### Gender inequality

Gender inequality was measured with the Gender Inequality Index (GII; United Nations Development Programme, 2021). In evaluating measures of global gender inequality, we considered indexes capturing the complex and multidimensional aspects of gender disparity, including the GII and the Gender‐related Development Index. Our decision to select GII over other measures of gender disparity was primarily based on a large body of cross‐national literature that utilized the GII to examine child outcomes (e.g., Brinda et al., 2015; Klevens & Ports, 2017; Marphatia et al., 2016).

The GII measures female disadvantage along three dimensions: reproductive health, the labor market, and empowerment. The reproductive health dimension is measured using the maternal mortality ratio and adolescent birth rate. The labor market dimension is measured via female and male labor force participation rates. The empowerment dimension is measured by examining the female and male population with at least a secondary education, and the female and male shares of parliamentary seats. To gather data along these dimensions, the United Nations Development Programme (UNDP) relies on publicly available international databases such as the WHO, UNICEF, World Bank, International Parliamentary Union, UN Department of Economic and Social Affairs World Population Prospects, International Labour Organization, and United Nations Population Funds. The UNDP uses these dimensions to create a country‐level composite measure that ranged from 0 to 1, with higher values indicating greater female disadvantage in the country. To increase the interpretability of these measures, the GII was multiplied by 100, which converted these measures into percentages (e.g., if GII was originally 0.212, the new value would be 21.2). The GII was downloaded from the UNDP Data Center website (http://hdr.undp.org/en/data) and merged with the UNICEF MICS data by country. The GII from the prior year of the household interview were used in the analysis. That is, if the household interview occurred in 2017, the GII from 2016 was used. If the GII from the prior year was unavailable, data from the closest available year were used.

#### Controls

Control variables were informed by prior studies that have examined discipline behaviors using UNICEF MICS data (Akmatov, 2011; Grogan‐Kaylor et al., 2021; Pace et al., 2019; Ward et al., 2021). Of note, UNICEF MICS does not measure race. While many MICS surveys collect data on respondents' ethnicity, there is immense ethnic diversity within and between countries in the analytic sample. Given there are many categories for ethnicity across the countries in MICS, we do not include a variable for this. However, we do account for country in the statistical models, which helps to account for ethnic diversity between countries. Household wealth quintile was standardized relative to the country's wealth score, and consisted of five categories: (1 = *poorest* [reference], 2 = *second poorest*, 3 = *middle*, 4 = *fourth poorest*, and 5 = *richest*). Number of household members was a continuous variable capped at 50. The cap of 50 was chosen due to very small *N*s (i.e., *n* < 20) that occurred after 50, and due to the difficulty of determining whether numbers higher than 50 were legitimate (e.g., two respondents stated they had a household size of 110). Age of the randomly selected reference child was continuous and measured in years (range: 1–17 years). Of note, the original survey design intended to measure parental discipline for children ages 1–14 years. However, some countries measured discipline up to age 17 years (*n* = 237 in our sample), which we kept in the analysis. Results do not change when these children are removed. Head‐of‐household sex and the sex of the randomly selected reference child were dichotomous (0 = *male*, 1 = *female*), as was whether the respondent was the child's biological parent (0 = *other caregiver*, 1 = *biological parent*). Mothers' and fathers' education were categorical (0 = *none* [reference], 1 = *primary*, 2 = *secondary‐plus*). Attitudes toward physical punishment were measured by asking the respondent whether they believed that children need physical punishment in order to be raised properly (0 = *no*, 1 = *yes*). Community type (0 = *rural*, 1 = *urban*) and MICS round number (0 = *Round 4*, 1 = *Round 5*) were dichotomous. GDP was retrieved from the Development Indicators provided by the World Bank (2023), and retrieved using the World Development Indicators package in R (Arel‐Bundock, 2022). GDP was divided by 1000 to increase interpretability.

### Statistical analysis

Multilevel logistic regression models using maximum likelihood estimation were conducted in Stata version 17.0 (StataCorp, 2021), with individuals nested within countries, and with a random intercept estimated for country. We did not use MICS sampling weights, as research suggests weighting can reduce estimated precision when individual‐level terms are clustered within a group, such as a state or country (Dickens, 1990; Friedman, 2013). Listwise deletion was used to handle missing data and resulted in an analytic sample of 126,165 households across 51 countries. Participants in the analytic sample had higher household wealth, more household members, had fathers with higher levels of educational attainment, had mothers who were less likely to have secondary education, were less likely to feel that children need corporal punishment, and were more likely to come from urban areas.

We first ran a main effects model, with the discipline items, GII, and control variables predicting child aggression. We then ran an interaction model, with GII interacting with each form of discipline. Our final statistical model was as follows:

$$\begin{array}{c} \text{logit}\left(y\right)=\beta_{0}+\beta_{1}\;\mathrm{GII}+\Sigma \;\beta_{k}\;{\text{discipline}}_{k}\\ \;+\Sigma \;\beta_{m}\;{\text{discipline}}_{k}\times \mathrm{GII}+\Sigma \;\beta_{n}\;{\text{covariate}}_{n}+\mu_{\mathrm{ij}}. \end{array}$$

Here, β_0_ was the intercept while β_1_ was the regression coefficient for gender inequality. The β_*k*_ were the regression coefficients for the *k* discipline items, the β_*m*_ were the regression coefficients for the interaction of each of these *k* discipline items with the GII. Σ β_*n*_ represented a set of *n* regression coefficients for the n covariates. *μ*_0*j*_ was a random intercept to indicate residence in each of the countries in our sample.

The coefficients for interaction terms (Σ β_*m*_) in logistic regression may not be truly indicative of the underlying differences in predicted probabilities, and indeed may provide misleading results (Ai & Norton, 2003; Karaca‐Mandic et al., 2012). We therefore probed the interaction effects by estimating the predicted probabilities of child aggression for children who had, and had not, experienced a particular form of discipline at the 10th, 25th, 50th, 75th, and 90th percentiles of the GII. We estimated pairwise comparisons of these predicted probabilities at each level of GII to assess the difference of predicted probabilities of children who had, and had not, experienced a particular form of discipline across the aforementioned percentiles of GII. We also calculated the difference of the differences in probabilities to test whether the strengths of associations differed at the GII extremes—specifically, we tested the differences in the strength of the association between discipline and aggression at the 10th and 90th percentiles of GII. By testing the statistical significance of differences in probabilities, rather than testing the statistical significance of coefficients of odds ratios, we tested what VanderWeele and Knol (2014) would term additive interactions.

## RESULTS

**FIGURE 1:**
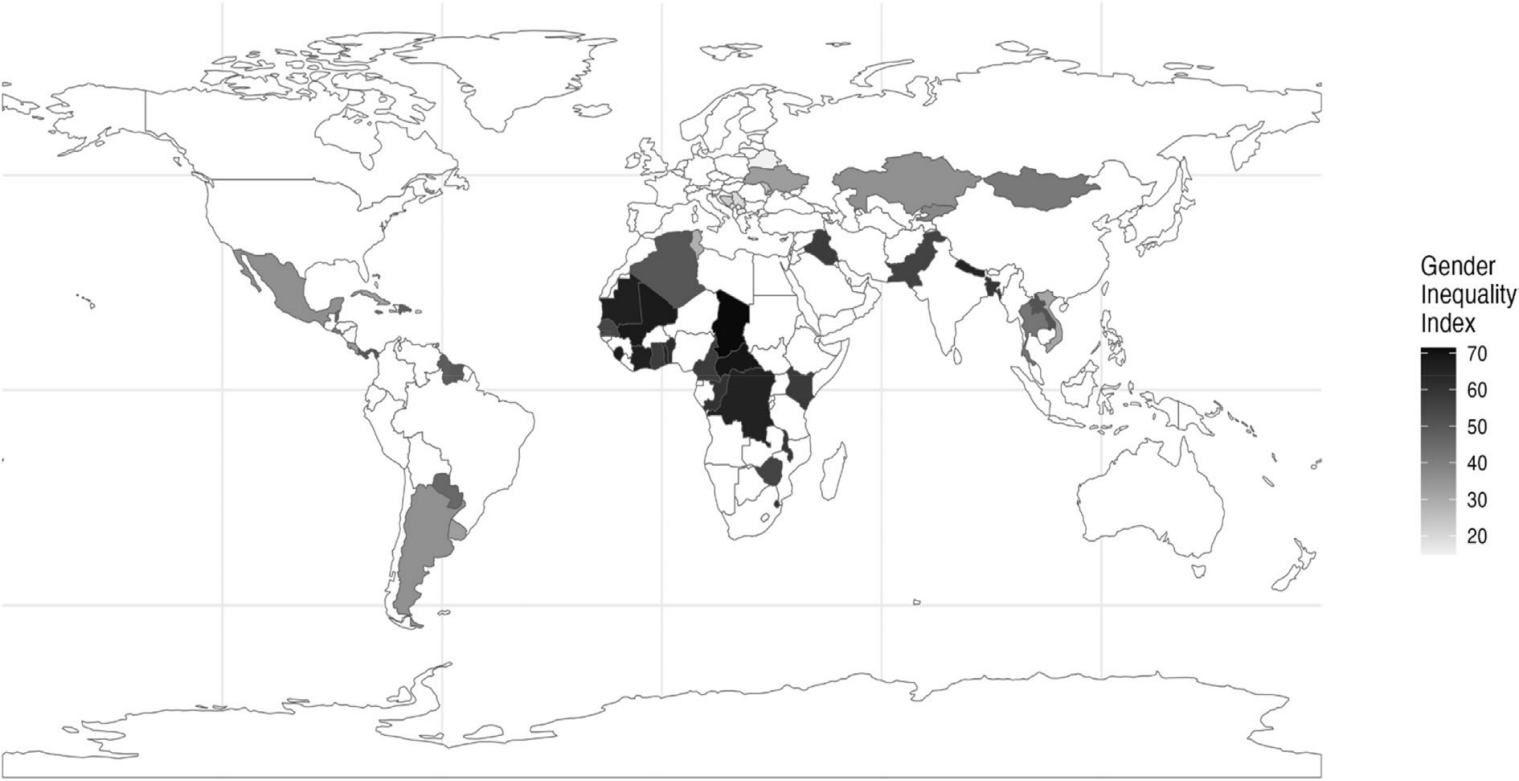
Gender Inequality Index scores across low‐ and middle‐income countries in the study sample.

Descriptive statistics of the sample can be found in Table 1. A visual depiction of countries and GII scores can be found in Figure 1. Our model did not yield any convergence issues. The unconditional intraclass correlation coefficient suggested that 8% of the variability in child aggression could be explained by the country in which the child was living.

**TABLE 1:** Descriptive statistics of study variables (*N* = 208,156 households, 63 countries).

	*N*	%	*M*	SD	Min	Max
Child outcome						
Aggression	80,145	38.50				
Psychological aggression						
Shouted at child	136,917	65.85				
Physical aggression						
Spanked child	89,079	42.84				
Beat child as hard as one could	11,706	5.63				
Nonaggressive behaviors						
Verbal reasoning	164,853	79.24				
Maternal education						
None	69,389	33.39				
Primary	62,722	30.18				
Secondary‐plus	75,684	36.42				
Paternal education						
None	41,186	25.11				
Primary	49,467	30.16				
Secondary‐plus	73,386	44.74				
MICS round						
Round 4	87,982	42.27				
Round 5	120,174	57.73				
Wealth quintile						
Poorest	54,640	26.53				
Second poorest	45,117	21.90				
Middle	39,459	19.16				
Fourth poorest	35,718	17.34				
Richest	31,055	15.08				
Reference child is female	103,126	49.54				
HH is female	35,189	16.91				
HH is biological parent	155,650	74.78				
HH believes children need physical punishment	67,400	32.93				
Urban community	85,631	41.14				
Reference child age (years)			5.65	3.41	1	17
Household members			6.65	3.49	2	50
Gender Inequality Index			52.22	12.78	14.9	71.4
Gross Domestic Product			3.41	3.09	0.3	17.9

*Note*: Number of household members was capped at 50. HH = survey respondent, typically the head of household.

Abbreviation: MICS, Multiple Indicator Cluster Surveys.

### Main effects

Results from the main effects model can be found in Table 2. GII and all four discipline items were significant predictors of child aggression. Specifically, a 1% increase in GII was associated with 3% higher odds of child aggression (OR = 1.03, *p* < .001). Further, shouting was associated with 31% higher odds of child aggression (OR = 1.31, *p* < .001); spanking was associated with 25% higher odds of child aggression (OR = 1.25, *p* < .001); and beating was associated with 36% higher odds of child aggression (OR = 1.36, *p* < .001). Verbal reasoning was associated with 3% lower odds of child aggression (OR = 0.97, *p* = .045). GDP, reference child age, head‐of‐household sex, and urban versus rural setting were not significantly associated with child aggression.

**TABLE 2:** Main effects model of discipline behaviors and Gender Inequality Index predicting child aggression (*N* = 126,165 households, 51 countries).

Variables	Aggression			
	OR	LL	UL	*p*
Gender Inequality Index	**1.03**	1.02	1.04	<.001
Gross Domestic Product	1.03	1.00	1.07	.060
Verbal reasoning	**0.97**	0.94	0.99	.045
Shouted at child	**1.31**	1.28	1.35	<.001
Spanked child	**1.25**	1.22	1.29	<.001
Beat child as hard as one could	**1.36**	1.29	1.45	<.001
Reference child age	0.99	0.99	1.00	.643
Selected child female	**0.88**	0.86	0.90	<.001
HH is biological parent	**0.95**	0.92	0.99	.018
HH is female	1.05	0.99	1.10	.107
Household members	**1.01**	1.01	1.02	<.001
Second poorest	0.98	0.95	1.02	.301
Middle	0.96	0.93	1.00	.060
Fourth poorest	0.97	0.93	1.01	.171
Richest	**0.93**	0.88	0.97	.003
Urban community	1.03	0.99	1.06	.062
MICS round 5	**0.83**	0.78	0.88	<.001
HH believes children need physical punishment	**1.09**	1.06	1.12	<.001
Mother primary education	1.02	0.99	1.06	.193
Mother secondary education	**0.93**	0.89	0.98	.002
Father primary education	**1.06**	1.02	1.10	.003
Father secondary education	1.00	0.96	1.04	.991

Abbreviations: HH, head of household; LL, lower limit of 95% confidence interval; MICS, Multiple Indicator Cluster Surveys; OR, odds ratio; UL, upper limit of 95% confidence interval.

Odds ratios where p < .05 are bolded.

### Interaction effects

**FIGURE 2:**
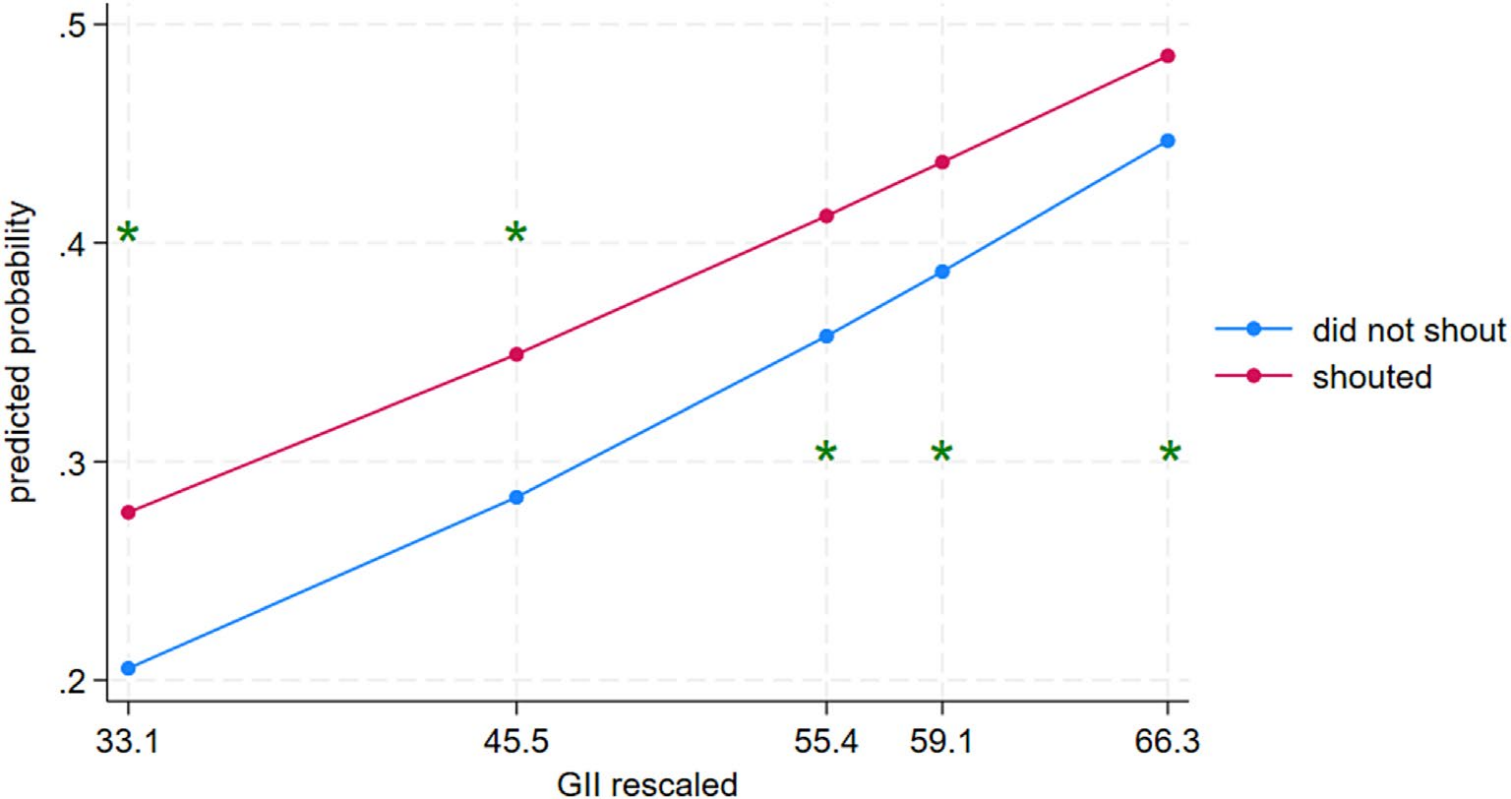
Interaction between gender inequality and shouting predicting child aggression. GII, Gender Inequality Index. *Statistically significant difference in probabilities across discipline groups in its corresponding percentile of GII, according to pairwise comparisons.

Results from the interaction effects model can be found in Table 3. All interaction effects were statistically significant. Parental shouting (see Figure 2) was significantly associated with child aggression at the 10th (contrast = 0.07, 95% CI [0.06, 0.09]), 25th (contrast = 0.07, 95% CI [0.06, 0.07]), 50th (contrast = 0.05, 95% CI [0.05, 0.06]), 75th (contrast = 0.05, 95% CI [0.04, 0.06]), and 90th percentiles (contrast = 0.04, 95% CI [0.03, 0.05]) of GII. Importantly, the association between shouting and child aggression was weaker when gender inequality was higher (i.e., the 90th percentile of GII) compared to when gender inequality was lower (i.e., the 10th percentile of GII) (contrast = 0.01, 95% CI [0.01, 0.05]).

**TABLE 3:** Interaction effects for Gender Inequality Index (GII) and parental discipline behaviors predicting child aggression (*N* = 126,165 households, 51 countries).

Interaction	Aggression			
	Ratio of odds ratios OR	LL	UL	*p*
GII × verbal reasoning	1.01	1.00	1.01	<.001
GII × shouted	0.99	0.99	0.99	<.001
GII × spanked	0.99	0.99	0.99	.023
GII × beat hard	0.99	0.98	0.99	.002

*Note*: All variables from the main effects model were included but are not shown in this table.

Abbreviations: LL, lower limit of 95% confidence interval; UL, upper limit of 95% confidence interval.

**FIGURE 3:**
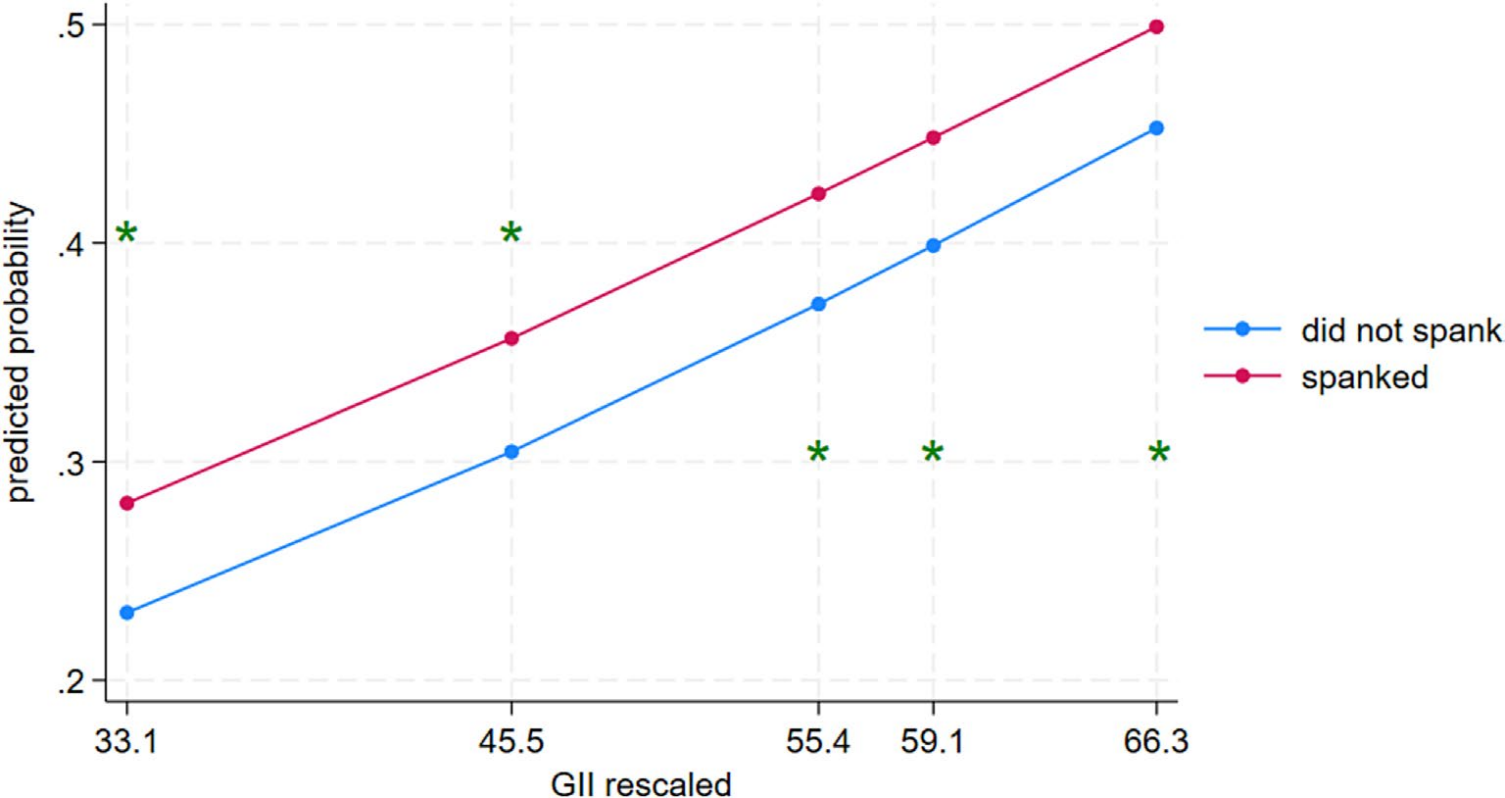
Interaction between gender inequality and spanking predicting child aggression. GII, Gender Inequality Index. *Statistically significant difference in probabilities across discipline groups in its corresponding percentile of GII, according to pairwise comparisons.

Parental spanking (see Figure 3) was significantly associated with child aggression at the 10th (contrast = 0.05, 95% CI [0.04, 0.06]), 25th (contrast = 0.05, 95% CI [0.04, 0.06]), 50th (contrast = 0.05, 95% CI [0.04, 0.06]), 75th (contrast = 0.05, 95% CI [0.04, 0.06]), and 90th percentiles (contrast = 0.05, 95% CI [0.04, 0.06]) of the GII. However, the association of spanking with child aggression did not differ between countries with lower or higher GII (contrast = 0.00, 95% CI [−0.01, 0.02]).

**FIGURE 4:**
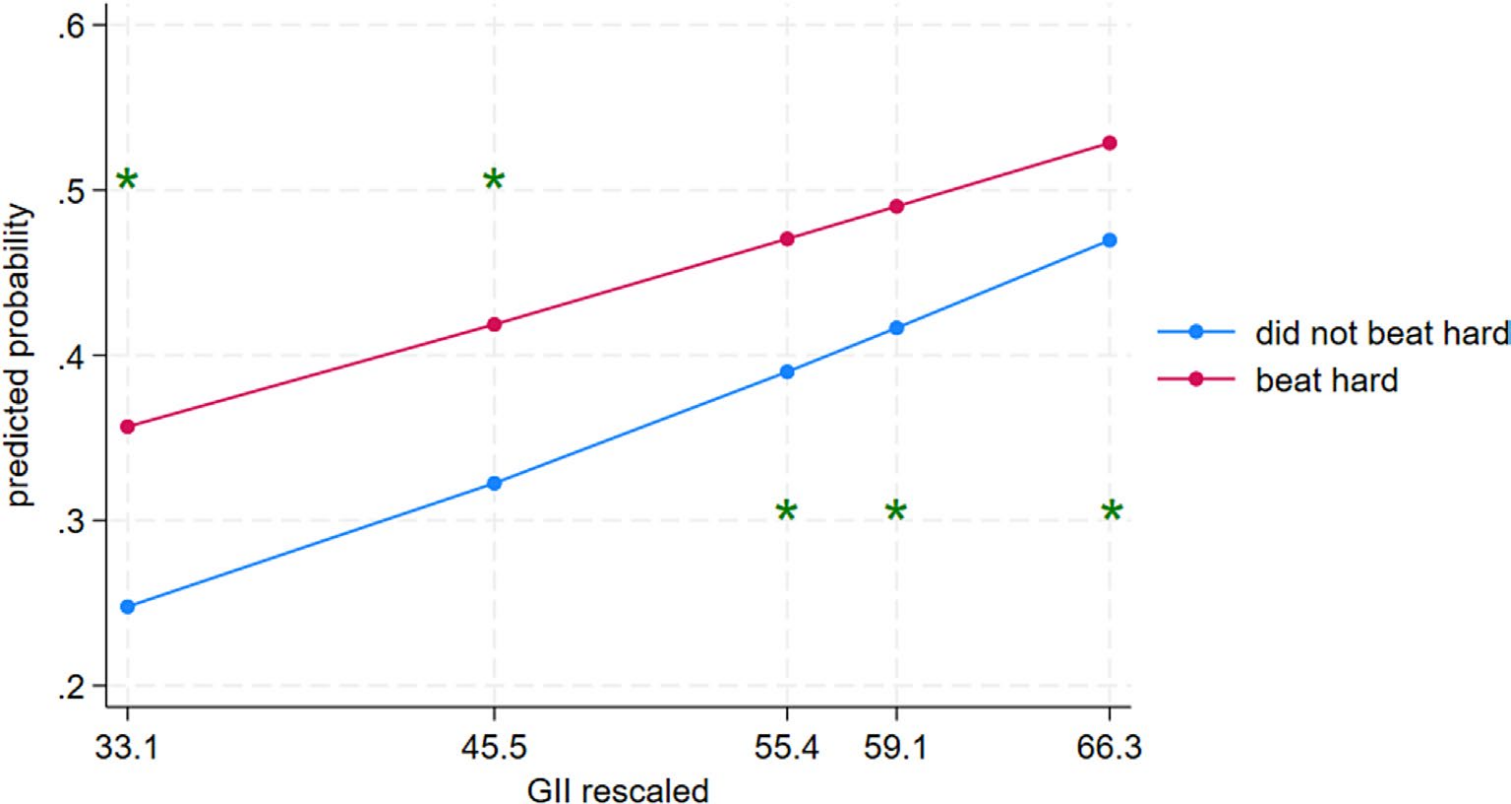
Interaction between gender inequality and beating predicting child aggression. GII, Gender Inequality Index. *Statistically significant difference in probabilities across discipline groups in its corresponding percentile of GII, according to pairwise comparisons.

Parental beating (see Figure 4) was significantly associated with child aggression at the 10th (contrast = 0.11, 95% CI [0.07, 0.15]), 25th (contrast = 0.09, 95% CI [0.08, 0.12]), 50th (contrast = 0.08, 95% CI [0.06, 0.09]), 75th (contrast = 0.07, 95% CI [0.06, 0.08]), and 90th percentiles (contrast = 0.06, 95% CI [0.04, 0.07]) of the GII. Importantly, the association between beating and child aggression was weaker when gender inequality was higher (i.e., the 90th percentile) compared to when gender inequality was lower (i.e., the 10th percentile) (contrast = 0.05, 95% CI [0.01, 0.10]).

**FIGURE 5:**
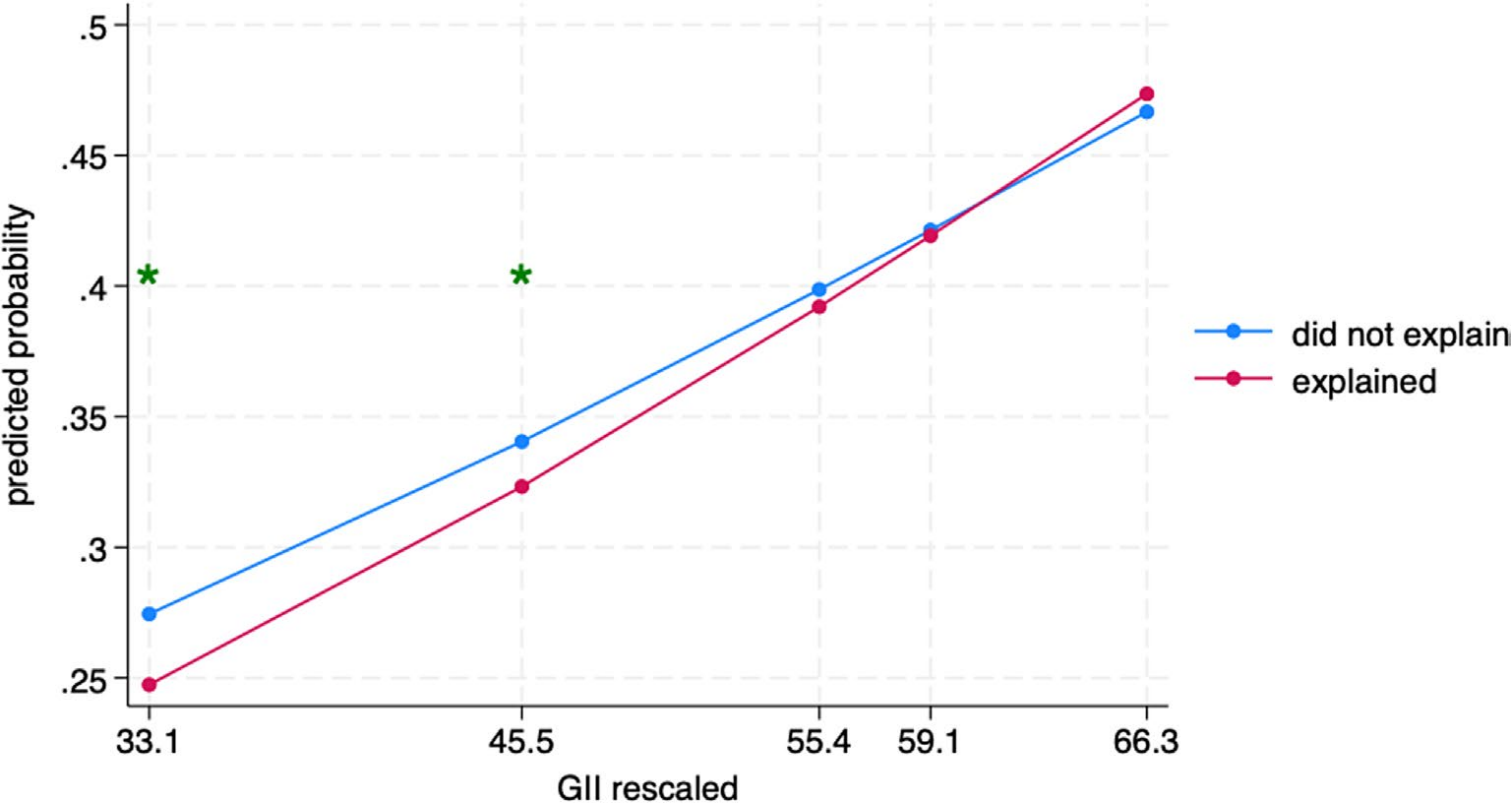
Interaction between gender inequality and verbal reasoning predicting child aggression. GII, Gender Inequality Index. *Statistically significant difference in probabilities across discipline groups in its corresponding percentile of GII, according to pairwise comparisons.

Parental verbal reasoning (see Figure 5) was significantly associated with child aggression at the 10th (contrast = −0.03, 95% CI [−0.04, −0.01]) and 25th percentiles (contrast = −0.02, 95% CI [−0.03, −0.01]), but was not significantly associated with child aggression at the 50th (contrast = −0.01, 95% CI [−0.01, 0.00]), 75th (contrast = 0.00, 95% CI [−0.01, 0.01]), or 90th percentiles (contrast = 0.01, 95% CI [0.00, 0.02]) of the GII. Importantly, the association between verbal reasoning and child aggression was weaker when gender inequality was higher (i.e., the 90th percentile) compared to when gender inequality was lower (i.e., the 10th percentile) (contrast = −0.04, 95% CI [−0.06, −0.02]).

## DISCUSSION

Previous research has shown the importance of the development of children's self‐control for increasing positive and reducing negative adult outcomes across multiple countries (Koepp et al., 2023; Moffitt et al., 2011). UNICEF recognizes the formative early years as a critical developmental period for child socio‐emotional development that requires targeted intervention in low‐resource settings. Indeed, prior studies demonstrate the importance of the proximal context of parenting and caregiving on child development index outcomes, including child socio‐emotional development (Bizzego et al., 2022; Bornstein et al., 2022). This study examined whether parental discipline behaviors interacted with country‐level gender inequality (i.e., female disadvantage in reproductive health, labor market participation, attaining a secondary education, and parliamentary representation) to predict child aggression in households with children between 36 and 59 months living in LMICs. Prior research has shown that gender inequality has deleterious effects on both male and female child development—with gender inequality being associated with lower educational attainment, higher rates of violence, and decreased labor market participation for women; higher gang violence, recruitment into armed forces, and child labor rates for men; and increased mental health problems and hygiene and sanitation concerns for men and women (Deuchar & Weide, 2019; Klasen & Lamanna, 2009; UNICEF, 2021). In order to understand how such rules and values of different countries may relate to parenting behaviors and subsequent child aggressive behavior, we examined the extent to which gender inequality interacts with parental discipline to predict child aggression at an early vulnerable age (36–59 months) among families in LMICs. We hypothesized that the relationship between discipline and child aggression would be weaker in contexts where gender inequality was higher.

Consistent with prior research (Nivette et al., 2014; Ward et al., 2023), results showed that child aggression was higher in countries with higher levels of gender inequality, and aggressive forms of parenting (i.e., shouting, spanking, and beating) were associated with higher probabilities of child aggression across levels of gender inequality. As a whole, consistent with the study hypothesis, results suggest that when gender inequality was lower (i.e., more gender parity), parental discipline had stronger associations with child aggression—for better or for worse. However, again consistent with the study hypothesis, when gender inequality was high, parental discipline had relatively weaker associations with child aggression. When country‐level gender inequality was lower (i.e., more gender parity), the power of positive parenting appeared to be unlocked, such that verbal reasoning was associated with lower probabilities of children being aggressive. Yet, as hypothesized, when there was higher country‐level female disadvantage, positive parenting had relatively weaker associations with child aggression.

Broadly speaking, the world values framework proposes that as country‐level support for gender equality increases, individual‐level behavior in those countries tends to be less constrained by gender roles than would be the case in countries with higher levels of gender inequality (Inglehart & Oyserman, 2004; Inglehart & Welzel, 2005). Our results provide support for this idea, as applied to the micro‐context interactions of parent–child discipline behaviors. Specifically, we found that in contexts where gender inequality was low, parenting behaviors (i.e., aggressive [shouting and beating] and nonaggressive [verbal reasoning]) exerted greater influence on child outcomes; however, in contexts where gender inequality was high, that is, where women experienced higher levels of disadvantage and less political and economic freedom, the macro‐level context of gender inequality exerted significantly more influence on children's outcomes. These results could be interpreted to be consistent with the notion that, as societies move toward higher levels of self‐expression and greater gender equality, gender roles may be less constraining in micro‐level contexts (Inglehart & Oyserman, 2004; Inglehart & Welzel, 2005).

Notably, contrary to our study hypothesis, the effects of parental spanking did not differ across levels of the GII. That is, regardless of the overall level of gender inequality within a country, parental spanking was associated with higher levels of child behavior problems. This aligns with previous studies in LMICs (Bizzego et al., 2022; Pace et al., 2019), other international locations (Gershoff et al., 2010), and in the United States (Gershoff & Grogan‐Kaylor, 2016; Grogan‐Kaylor et al., 2020; Lee et al., 2020; Ward et al., 2020) that have found consistent associations of physically aggressive parenting and spanking with child aggression, regardless of contextual factors.

Our main effect analyses did not find a statistically significant association between reference child age and focal child aggression, which differs from prior research of primarily Western samples suggesting nonlinear associations between child age and aggression (Tremblay et al., 2004). However, a recent analysis among a large sample of children in LMICs (Ward, 2022) suggests that the nonlinear association between child age and aggression is moderated by parental discipline. Specifically, when parents in LMICs abstained from aggressive discipline techniques, child aggression decreased over time; however, when parents in LMICs used harsh discipline techniques (i.e., shaking, hitting with an object), child aggression did not decrease over time.

It is also interesting that our results suggested country‐level GDP was not associated with child aggression (even though the GII was associated with aggression). One possibility is that gender inequality may mediate—or statistically explain—the connection between GDP and aggression. Indeed, GDP becomes statistically significant when GII is removed from the model. However, we are mindful of literature that suggests that possible mediation relationships are statistically indistinguishable from relationships where there are correlated regressors (O'Laughlin et al., 2018). Lastly, it is possible that some unobserved confounder—perhaps an unobserved dimension of country level inequality—may predict both GDP and GII, with a subsequent effect on child aggression. A final possibility is that, due to the fact that our sample of countries are all from LMICs, there may not be enough variation in GDP to demonstrate a statistically significant association with our outcome of interest. Without strong theoretical rationale to suggest a preference for one explanation over another, we suggest that all have at least some plausibility.

### Implications

These results suggest that there is a lower probability of child aggression when positive forms of parenting are being used in contexts where country‐level gender inequality is low. From an intervention standpoint, this could suggest that, for positive parenting interventions to improve child developmental outcomes in a country with high gender inequality, efforts may be needed to improve country‐level women's reproductive health (i.e., maternal mortality and adolescent birth rate), labor force participation, attainment of secondary education, and parliamentary representation. This also suggests that, while solely providing parenting interventions in countries where gender inequality is low may result in positive child outcomes, administering micro‐level interventions alone in countries where gender inequality is high may not result in substantial changes in child development, unless these interventions are coupled with macro‐level interventions that increase women's empowerment.

The study findings could inform new ways of applying ecological and cultural change perspectives to low‐resourced settings. Building upon existing theoretical frameworks that center marginalized populations (García Coll et al., 1996), our study proposes that broader societal‐level inequities may dampen parental opportunity for micro‐level impact on child development. This is not to say that parenting is unimportant in gender inequitable, low‐resourced settings; rather, the relative potential for parents to be able to substantially change a child's socio‐emotional trajectory may be weakened. This supposition gives credence to the importance of the “self‐expression” dimension of cross‐cultural variation, which promotes humanistic and emancipative human ethos and a shift toward gender equality (Inglehart & Welzel, 2005). We build upon this thinking by proposing that, as societies move more toward “self expression” values, parenting will have more salience and power to change child developmental trajectories–for better (in the case of positive parenting) and for worse (in the case of aggressive and abusive parenting). We theorize that as societies grant more power to women, they will, in turn, be granting more power to parents.

### Limitations

The results of this study should be interpreted while considering its limitations. The gender inequality variable was a large index with multiple dimensions, namely maternal mortality, adolescent birth rate, female secondary education attainment, female parliamentary representation, and female labor force participation. Although the incorporation of the various dimensions within the GII is mathematically useful, it may lack interpretative specificity. Future research could examine specific dimensions of the GII. Relatedly, the size of many parental discipline‐macrosystem interactions were relatively small. This is partially because the GII was entered into the model as percentages (i.e., the regression captures a 1% increase in GII); therefore, referencing the graphs of the interactions is needed to better understand the magnitude of the interactions. Further, as macro–micro interactions are difficult to capture generally, future research will need to replicate these analyses to determine under what circumstances GII may meaningfully affect how parenting relates to child outcomes. It is important to note that, with our large sample size, even small effects are often statistically significant. Nevertheless, micro–macro interactions are difficult to detect quantitatively, and necessitate a large sample size.

Because the data were cross‐sectional, all interpretations are limited to associations. This means that the directionality of associations may be reversed, wherein poor child socio‐emotional behaviors precede disciplinary action. Appropriate longitudinal research designs while employing analytic methods such as fixed‐effects regression (which helps with ruling out the effects of unobserved confounders) and cross‐lagged models (which helps establish directionality of associations) will be needed to strengthen researchers' ability to make intervention recommendations. Additionally, this study only analyzed households with a focal child aged 3–4 years old, and a reference child aged 1–17 years old, and cannot provide insight into associations between parenting behaviors and child outcomes among households with only older children.

Nearly all variables used in this study are based on self‐report data, which may have been susceptible to social desirability bias and self‐presentation bias (potentially resulting in parents failing to report using aggressive forms of discipline), missing data bias, or other forms of inaccurate reporting such as difficulty recalling parenting behaviors that occurred in the past month (Memmott‐Elison et al., 2020). Further, the parental discipline variables were dichotomous, which precludes the ability to capture the frequency or severity of discipline behaviors. Relatedly, the child outcome variable was also dichotomous, which means the results cannot speak to the severity of child aggression.

## CONCLUSION

Guided by the bioecological model of human development, this study examined how microsystem (i.e., aggressive and nonaggressive forms of parental discipline) and macrosystem (i.e., gender inequality) factors interact to predict child aggression in LMICs. Results revealed that aggressive forms of discipline (shouting, spanking, and beating) were associated with higher probabilities of child aggression across all levels of gender inequality; however, verbal reasoning was only associated with lower probabilities of child aggression in contexts when gender inequality was low. Further, across all forms of discipline except spanking, associations between discipline with child aggression were *weaker* in countries where gender inequality was *higher*. This study underscores the importance of understanding the role of reducing aggressive discipline and increasing the use of nonaggressive discipline—specifically verbal reasoning—in low‐resourced contexts. This study also suggests that, for parenting to be strongly associated with child outcomes in LMICs, gains will need to be made in country‐level gender parity. Cross‐sectoral interventions that address the parent–child relationship and the macro‐level social context may be an important avenue for improving child development in LMICs.

## Data Availability

The data necessary to reproduce the analyses presented here are publicly accessible. The analytic code necessary to reproduce the analyses presented in this paper is available by contacting the first author. The materials necessary to attempt to replicate the findings presented here are publicly accessible. The analyses presented here were not preregistered.
